# Phage biocontrol success of bacterial wilt depends on synergistic interactions with resident rhizosphere microbiota

**DOI:** 10.1111/1751-7915.70049

**Published:** 2024-11-13

**Authors:** Sara Franco Ortega, Bryden Fields, Daniel Narino Rojas, Lauri Mikonranta, Matthew Holmes, Andrea L. Harper, Ville‐Petri Friman

**Affiliations:** ^1^ Department of Biology University of York York UK; ^2^ Centre for Novel Agricultural Products, Department of Biology University of York York UK; ^3^ Present address: Fera Science Ltd., York BioTech Campus Sand Hutton York UK; ^4^ Present address: Department of Microbiology University of Helsinki Helsinki Finland

## Abstract

Phages can successfully be used in vitro and in planta to biocontrol the phytopathogenic *Ralstonia solanacearum bacterium*—the causal agent of bacterial wilt disease. However, phage biocontrol outcomes are still variable, and it is unclear what causes this. In this study, we assessed the efficiency of four phages in controlled in vitro and in planta experiments in all one‐ and two‐phage combinations. We found that using phages in combination did not improve the phage biocontrol efficiency relative to single phage treatments, while certain phages and their combinations were more effective than the others. High intra‐treatment variability in phage efficiency was observed across all phage treatments, which was associated with clear shifts in microbiome composition, a reduction in *R. solanacearum* and an increase in phage densities. We further identified the bacterial taxa that were associated with these ‘shifted’ microbiomes and conducted additional plant growth experiments, demonstrating that some of the enriched bacterial species could protect plants from *R. solanacearum* infections—a pattern which was also observed using partial least squares path modelling (PLS‐PM). Together, these results suggest that phages could open niche space for beneficial bacteria by reducing pathogen densities and that variability in phage biocontrol outcomes is rhizosphere microbiome‐dependent, which can introduce between‐replicate variation, even in controlled greenhouse conditions.

## Graphical abstract

Phage biocontrol success against the major plant pathogenic bacterium *Ralstonia solanacearum*, causal agent of bacterial wilt disease in tomatoes, depends on interactions with rhizosphere microbiome.

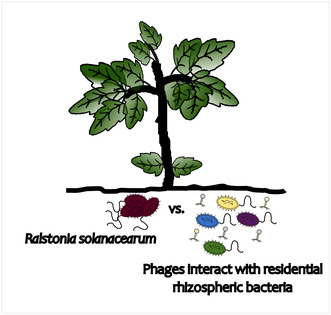

## INTRODUCTION

It is expected that the human population will reach approximately 10 billion people by 2050, resulting in an 80%–110% increase in the demand for food. To achieve this, it is crucial to reduce global losses to plant pathogens (Strange & Scott, 2005), especially in the face of climate change, which could affect pathogen virulence and disease development (Singh et al., 2023). *Ralstonia solanacearum* is one of the most destructive bacterial phytopathogens worldwide (Mansfield et al., 2012; Wang, Luo, et al., 2023), being a major threat to agriculture and food security. It can infect more than 250 plant species and is especially deadly to solanaceous plants, causing brown rot in potatoes and bacterial wilt disease in tomatoes (Elphinstone, 2005). Unfortunately, we still lack effective control measures against *R. solanacearum*, as traditional agronomic practices such as crop rotation and grafting (Lemaga et al., 2001), or chemical control methods (Nicolopoulou‐Stamati et al., 2016), have proven ineffective. Novel, effective and environmentally friendly control methods are hence urgently required.

During the last decade, microbial biocontrol methods have shown promise in controlling *R. solanacearum* (Álvarez et al., 2019; Hu et al., 2021; Narasimha Murthy et al., 2021; Wei et al., 2017). These methods are often based on pathogen suppression by antagonistic microorganisms, such as bacteria and fungi, that compete for shared resources with the pathogen or produce antimicrobials that suppress pathogen growth (Beneduzi et al., 2012; Hu et al., 2021; Narasimha Murthy et al., 2021). Recently, the use of pathogen‐specific viruses, bacteriophages (phages for short), to biocontrol phytopathogenic bacteria has received more interest (Cao et al., 2018; Wamani et al., 2023; Yamada et al., 2007; Nion et al., 2015). Phages are efficient at killing bacteria (Wang et al., 2019, 2024), which could provide additional time for the plant host immune system to combat the pathogen infection. Moreover, phages are self‐replicating and self‐limiting, only increasing their abundance when their host bacterium is present. Despite these advantages, phage biocontrol outcomes are variable (Hong et al., 2014; Wang et al., 2019, 2024; Wang, Wang, et al., 2023), and it is not clear how phages should be applied to achieve more consistent biocontrol efficacy.

Previous research has shown that the use of phage combinations and repeated application of phage can attain more efficient phage biocontrol results (Kalpage & De Costa, 2015; Wang et al., 2017, 2019) and improve *R. solanacearum* density control in the rhizosphere. Furthermore, it has been shown that phages can have synergistic effects with rhizospheric bacteria, resulting in improved *R. solanacearum* suppression when applied together (Wang et al., 2017, 2019, 2024). While such phage‐bacteria synergies have previously been linked with increased phage application frequency (Wang et al., 2024), it is unclear if interactions with the existing rhizosphere microbiota depend on the identity of phages or whether phages are applied individually or in combinations.

In this work, we tested if phage combinations are more effective at controlling bacterial wilt disease than single phages and if the *R. solanacearum* suppression could be linked with shifts in the suppressiveness of resident rhizosphere bacterial microbiota. In total, we used four phages that originated from the United Kingdom and China and applied them in all single‐phage and two‐phage combinations to control the *R. solanacearum* strain UW551 in the lab and greenhouse experiments (only 2‐phage combinations were used to keep the experimental design manageable). In addition to tracking bacterial wilt disease progression, we analysed changes in the bacterial community diversity and composition at the end of the greenhouse experiment and directly tested if any of the enriched rhizobacterial taxa showed pathogen suppression in vitro and in planta. Together, our results suggest that interactions between biocontrol phages and resident rhizosphere bacteria can result in variation in phage biocontrol outcomes.

## EXPERIMENTAL PROCEDURES

### Bacterial and phage strains and preparation of stock cultures

We used *R. solanacearum* UW551 strain as the model pathogen in this study, which was ordered from the National Culture Collection of Plant Pathogenic Bacteria (https://www.fera.co.uk/ncppb). Four phages were selected from the University of York collection for this study (Table 1). Three phages were isolated from the UK river water samples using *R. solanacearum* strain UW551 as the host bacterium; *R. solanacearum* and its phages are naturally found in the UK rivers (Elphinstone & Matthews‐Berry, 2020). In addition, one phage originating from a tomato field in China was used in this study (Table 1). Bacterial stocks were prepared by growing the bacteria in liquid CPG media (casamino acid 1 g/L, peptone 10 g/L, glucose 5 g/L, Merck, Gillingham, UK) for 72 h at 28°C with shaking (100 rpm) and preparing glycerol stocks (30% glycerol) that were stored at −80°C. Phage stocks were prepared by adding 60 μL phage supernatant stored at 4°C and 3 mL of UW551 bacterium at OD = 0.2, to 600 mL CPG into 1 L Erlenmeyer flasks. The cocultures were grown for 96 h at 28°C with shaking (100 rpm), centrifuged to pellet the bacteria (10 min at 5000 G) and the supernatant filtered through 0.2 μm filters to collect bacteria‐free phage lysate for the experiments (stored at 4°C in glass vials).

**TABLE 1:** Phages used in this study, including geographical location, year of isolation and mash dissimilarity distance between pairwise phage combinations.

Name	Collection/survey	Isolated from	Year	Dissimilarity with other phages			
				Phage 2	Mash distance	*p*‐Value	Shared‐hashes
PY04	York phage collection	River Thames, UK	2019	PY045	1	1	0/1000
				PY059	1	1	0/1000
				PY065	0.00134	0	946/1000
PY045	Nanjing phage collection	Tomato rhizosphere, Nanjing, China	2018	PY04	1	1	0/1000
				PY059	1	1	0/1000
				PY065	1	1	0/1000
PYO59	York phage collection	River Foss, UK	2021	PY04	1	1	0/1000
				PY045	1	1	0/1000
				PY065	1	1	0/1000
PYO65	York phage collection	River Jubilee, UK	2021	PY04	0.00134	0	946/1000
				PY045	1	1	0/1000
				PY059	1	1	0/1000

### Phage DNA extraction, genome assembly and annotation

The phage DNA isolation kit (Norgen, Biotek corporation, Thorold, ON, Canada) was used to extract phage DNA for sequencing, following the manufacturer's instructions with the following modifications: 15 μL of 20 units Norgen DNase I was added to the phage supernatants and incubated at room temperature for 30 min; DNase I was inactivated by incubating at 75°C for 10 min; 10 μL of 20 μg/mL proteinase K was added to the phage supernatants and incubated for 1 h at 55°C; phage supernatants were incubated with lysis buffer at 65°C for 30 min; DNA was eluted into 50 μL nuclease‐free water. A Qubit high sensitivity dsDNA kit was used to measure the amount of extracted DNA. The samples were sequenced by MicrobesNG services using Illumina Novaseq 6000 with a 250‐bp paired‐end strategy. Raw reads were trimmed by MicrobesNG using Trimmomatic (Bolger et al., 2014) and the quality was assessed using in‐house scripts combined with the following software: Samtools, BedTools and bwa‐mem (Li & Durbin, 2009). Phage genomes were assembled following the pipeline outlined in Shen and Millard (2021) and Turner et al. (2021). Briefly, Cutadapt (v2.3) (Martin, 2011) was also used to trim reads with less than 100 bp, and fastqc (v0.11.4) was used to check the read quality. After that, Seqtk (v 1.3; Li, 2023) was used to subsample paired‐end reads, so that genome assemblies would have around 100× coverage. Phage genomes were then subsequently assembled with the subsampled reads using SPAdes (Prjibelski et al., 2020). Appendix S1, Tables S1 and S2 include more information regarding the phage genome assembly. Raw data and genome assemblies are available in NCBI Sequence Read Archive (SRA) BioProject PRJNA1076092.

### Analysing genetic differences between phages based on MASH distance and orthologous genes

Genetic distance between the four phages was analysed by MASH using sketch and triangle to find the distance based on pairwise comparisons. Amino acid sequences were used in the Synteny Imaging tool (SYNIMA) (Farrer, 2017) pipeline using Orthofinder. After that, kinfin (version 3 [Laetsch & Blaxter, 2017]) was used to calculate the number of orthogroups shared between the different phages, which were visualised using the R package UpSetR. Based on mash distances, high similarity between PY04 and PY065 phages was found (0.00134, Table 1), while the rest of the pairwise comparisons showed mash distances of 1, indicating that these phages were completely different from each other based on the genome sequence data. Further SYNIMA analysis suggested that a missing HNH protein gene might affect the packaging and biocontrol activity of PY059 (See Text S1 for more detailed genome comparisons between the phages).

### Testing phage infectivity in vitro

To quantify the infectivity of phages on the UW551 host, both spotting and liquid assays were performed using all four phages individually (Wang et al., 2019). Liquid cultures were set up in a 96‐well plate by inoculating 170 μL of fresh CPG with 20 μL *R. solanacearum* OD = 0.1 (1E+02 CFUs/mL) and 10 μL of phages at 1E+06 PFUs/mL. Plates were incubated for 72 h at 28°C without shaking, and bacterial growth was measured as optical density (OD_600_ nm) at 2‐h intervals. Phage infectivity was calculated by measuring bacterial growth inhibition by the phage relative to bacteria‐only cultures (UW551 + 10 μL of sterile H_2_0), using area under the growth curves derived from the whole growth curve.

### Construction of phage combination treatments for bacterial wilt biocontrol experiments in planta

To compare the biocontrol efficiency of the phages, we tested the efficacy of each phage individually and in pairwise combination with all others, resulting in a total of four individual and six pairwise phage treatments. We used the *Solanum lycopersicum* (tomato) variety Moneymaker (Moles Seeds, Colchester, UK) in all plant experiments, due to its high susceptibility to *R. solanacearum* (Ji et al., 2021). Tomato seeds were sterilised in 1% bleach and rinsed with abundant water, after which they were directly grown in pots (approximately 51 × 48 × 50 mm) with approximately 120 g of John Innes No. 2 compost (free of *Ralstonia*) for 3 weeks at 24–26°C (16 h light/8 h dark—with lights on when ambient light intensity went below 90 W.m^−2^ and off if ambient light intensity went above 250 W.m^−2^) at the quarantine glasshouses at Fera Science Ltd. (York, UK). The plants were watered daily except for the day before UW551 inoculation. The plants were inoculated with *R. solanacearum* strain UW551 after 3 weeks of growth, and phages were added to the phage treatment plants 1 day after the bacterial inoculation. The *R. solanacearum* strain was prepared for inoculation following the protocols reported by Khokhani et al. (2018). First, we used CPG to grow the bacterium at 28°C for 72 h (100 rpm). One millilitre of culture was inoculated into 400 mL fresh CPG and maintained at 28°C for a further 24 h (100 rpm). After this, another 150 mL of fresh CPG was added, and cultures were grown for another 24 h in the same conditions. The OD_600_ of the final bacterial culture was adjusted to 0.7 (approximately 8E+09 cells/mL; the pellet was not washed before application), and a total of 5 mL of bacterial culture was inoculated onto each plant via root drench by applying the inoculum close to the plant. The phage inoculum was prepared as described previously and diluted to the concentration of 1E+06 PFU/mL using sterile water. In the case of 1‐phage treatments, 5 mL of each phage solution was used, and with 2‐phage treatments, 2.5 mL of each phage was used to keep the total phage numbers the same between the treatments (therefore, a total phage volume of 5 mL was applied to all phage treatments). The bacterium‐only treatment was inoculated with 5 mL of sterile water instead of phage inoculum (UW551‐only) and the negative control with no microbial inoculum received 5 mL of CPG (simulates bacterial inoculation effect) followed by 5 mL of sterile water (simulating phage inoculation). A total of 12 independent tomato plants were used for every treatment (all plants were grown individually on separate trays). Disease progression was measured at the onset of disease, from 10 days post‐inoculation (dpi) to 21 dpi, using the disease index score (DI), which varies on a scale from 0 to 4, where 0 denotes healthy plants without symptoms, while indexes between 1 and 4 were used for plants with bacterial wilt symptoms present on 25%, 50%, 75% and 100% of the aerial parts, respectively.

### Rhizosphere soil collection for microbial community analysis at the end of the greenhouse experiment

To assess the indirect effects of phages on soil rhizosphere bacteria, we explored bacterial species diversity and community composition at the end of the greenhouse experiment. The rhizosphere samples were collected at 21 dpi and processed on the same day: Plants were cut as close to the soil as possible, and the soil of the whole pot was homogenised. For each sample, approximately 2 g of the soil was stored at −80°C and were later used for DNA extraction using method 3 of the protocols described previously (Porteous & Armstrong, 1991; Tien et al., 1999). DNA concentrations were assessed with Nanodrop and stored at −80°C until further processing. Additional 2‐g subsamples of extracted soils were stored as 30% glycerol stocks for bacterial isolation, while another 2‐g subsamples were homogenised in 20 mL of sterile water, vortexed for 3 min and allowed to settle for 30 min to obtain soil washes. Ten millilitre of the soil washes were passed through a 0.2 μm filter (Sarstedt, Nümbrecht, Germany) and stored at 4°C for quantification of phage densities.

### Quantifying phage densities in rhizosphere soil samples

Phage densities were quantified as PFU/mL using the double agar overlay plaque assay (Kropinski et al., 2009). After diluting samples on 96‐well microplates using three technical replicates per sample, 300 μL of UW551 bacterial cultures and 180 μL of all diluted phage suspensions were mixed with 10 mL of warm CPG soft agar. The suspensions were mixed by gently inverting the tubes six times; then, they were immediately poured over a round CPG agar plate and left to solidify. Plates were incubated at 28°C for 24 h, and subsequently, phage densities were determined as PFU/mL based on the number of visible plaques observed on the agar plates (Table S3).

### Rhizosphere bacterial community analysis

To compare changes in bacterial community composition and diversity between treatments at the end of the greenhouse experiment, 16S rRNA sequencing was performed by Novogene using a PE250 strategy on an Illumina NovaSeq6000 using the primers Forward GTGCCAGCMGCCGCGGTAA and Reverse GGACTACHVGGGTWTCTAAT targeting V4 region of the 16S ribosomal RNA. After first assessing the product sizes via PCR and gel runs, the same amount of PCR product was pooled for each sample, end‐repaired, A‐tailed and ligated to Illumina adapters. The library preparation and initial bioinformatic analyses were prepared by Novogene, where the raw data were spliced, and filtered to obtain clean sequence data. DADA2 (Callahan et al., 2016) was used to reduce noise and to obtain the final amplicon sequence variants (ASVs). Each ASV was then annotated to obtain species information using the SILVA database (Quast et al., 2013) and QIIME2's classify‐sklearn algorithm (Bokulich et al., 2018), using a pretrained Naive Bayes classifier. Each ASV was annotated at the kingdom, phyla, class, order, family, genus and species levels. Raw data are available in NCBI Sequence Read Archive (SRA) BioProject PRJNA1010659. We obtained an average of 119,002 raw read pairs for the 154 samples (after removing PY04 1‐phage treatment and one from the PY04 + PY045 2‐phage treatment due to poor quality) with an average length of 253 nucleotides, a GC content of 53.51%, Q20 of 99.55% and Q30 of 98.22% (Table S4). To obtain rhizosphere bacterial density estimates based on sequence data, the amount of extracted DNA was normalised with the weight of the soil used for the DNA extraction per sample. Absolute abundance was calculated (Table S5) and the taxonomy assignment of the ASVs was represented in Table S6. Pathogen abundances between treatments were calculated after rarefying the data by grouping together the two ASVs (ASV1 and ASV240) identified as *Ralstonia* (Tables S7 and S8).

A Phyloseq object was created using the package phyloseq in R using the normalised absolute abundance, the taxa file and the metadata (total of 55,575 taxa and 154 samples). Rarefaction was performed using the function rarefy_even_depth after filtering (2747 taxa). To separate phage effects from other variables, we also sequenced the bacterial communities from the negative control plant replicates (*N* = 12) which were not treated with *R. solanacearum* or phages and positive control plants that were inoculated with *R. solanacearum* only (*N* = 12).

### Random forest analysis to identify bacterial taxa in shifted rhizosphere microbiomes

After observing a shift in the bacterial microbiome composition in some of the plant replicate samples, we identified the specific taxa associated with these shifted communities. To achieve this, we performed a random forest analysis using the data of the samples treated with phages (1‐ and 2‐phage treatments, for a total of 118 plants, not including negative or positive controls) and categorised them into ‘shifted’ and ‘centred’ samples according to their position in the NMDS1 axis (as explained in the next section). The analysis was performed using the rarefied absolute count data (2747 taxa). The training set was created randomly by sampling 70% of all samples (80), while the remaining samples were used as the test data set to assess how well the model predicted the data (38 samples). The function randomForest from the package randomForest in R was used with default parameters, proximity = TRUE and 1000 trees. Average Mean Gini values were calculated for each bacterial genus to evaluate their relative importance in explaining the shift in microbiome composition.

### Isolation of bacteria from the end‐point rhizosphere samples and assessment of their biocontrol activity

To experimentally validate if bacteria associated with shifted microbiomes could explain the suppression of *R. solanacearum*, 100‐fold dilutions of soil washes derived from both diseased and healthy plants (Table 2) were plated onto TSA (tryptone‐soy agar), CPG agar and SMSA media which is a semi‐selective medium for *R. solanacearum* (Elphinstone et al., 1996). Plates were incubated at 28°C for 48 h, and single colonies streaked onto new media to isolate individual bacteria. Colony‐PCR was performed to identify the genera of >100 colonies by transferring a loop of the colony to 100 μL of sterile water, which was then boiled for 10 min and centrifuged before performing PCRs. The 16S rRNA sequence was amplified using the same primers that were used for the metabarcoding, and PCR products were purified using the Wizard® SV Gel and PCR Clean‐Up System (Promega, Southampton, UK) following the manufacturer's instructions. The products were quantified using a Nanodrop 2000, and Sanger‐sequenced using the forward primer. Based on the high Gini values obtained in the RF analysis, we focused on the following bacterial genera: four isolates assigned to *Burkholderia‐Caballeronia‐Paraburkholderia* sp., one *Bacillus* sp. isolate, two *Pseudomonas* sp. and two *Rhodanobacter* sp. isolates (Table 2). Despite several attempts, we failed to isolate *Sphingomonas* bacteria from the samples even though it was one of the most abundant taxa in the shifted microbiomes based on the sequencing data.

**TABLE 2:** Bacteria isolated from the rhizosphere soil and tested for their potential biocontrol activity against *R. solanacearum* using spotting agar and liquid co‐culture assays.

		Sample isolated ID	Phage treatment	Disease index at 21 dpi in the sample	Microbiome
B12	*Bacillus* sp.	FERA.105	PY045.PY065	0	Centred
P19	*Pseudomonas* sp.	FERA.1	PY04	4	Shifted
P91	*Pseudomonas* sp.	FERA.1	PY04	4	Shifted
R55	*Rhodanobacter* sp.	FERA.5	PY04	0	Centred
R68	*Rhodanobacter* sp.	FERA.31	PY059	4	Centred
B12	*Burkholderia* sp.	FERA.19	PY045	4	Shifted
B14	*Burkholderia* sp.	FERA.19	PY045	4	Shifted
PB10	*Paraburkholderia* sp.	FERA.13	PY045	0	Centred
PB18	*Prarburkholderia* sp.	FERA.25	PY059	0	Centred

To test if these isolated taxa had a positive or negative effect on *R. solanacearum*, in vitro and in vivo assays were performed. We first assessed the direct inhibition of *R. solanacearum* growth with a spot assay using a method similar to phage density measurements; instead of spotting phages, four technical replicates of 10 μL of selected bacterial isolates (Table 2) were inoculated on top of *R. solanacearum* lawns and pathogen inhibition was quantified as the diameter of inhibition halos observed on the plates. Liquid assays were also performed using a GFP‐tagged *R. solanacearum* UW551 strain obtained by transformation with the pCOMP‐PhII to introduce the recombination regions of the pRC plasmid, and then, the p‐COMP transformed colonies were used for a second transformation with pRCG‐Pps‐GFPuv (Cruz et al., 2014; Monteiro et al., 2012). In these assays, *R. solanacearum* growth was measured on 96‐well plates alone or in the presence of each of the 9 isolated rhizosphere taxa. All the strains were initially grown in CPG media for 48 h at 28°C with shaking (100 rpm) and then diluted to an OD = 0.1. Monocultures were prepared by inoculating 20 μL of bacteria cultures with 180 μL of CPG while cocultures with 20 μL of each bacterium were inoculated in 160 μL CPG (*N* = 3 replicates per treatment). Pathogen relative density was assessed based on GFP fluorescence signal intensity at regular intervals for 48‐h growth period (excitation at 405 nm and emission at 509 nm).

The potential biocontrol effects of a subset of bacterial taxa were tested in planta (*Pseudomonas* P19, *Rhodanobacter* R55, *Burkholderia* B12, *Burkholderia* PB18). Plants were grown for 3 weeks at 20°C (±2°C) with 14 h light/10 h dark cycle and moved to PHCbi growth cabinets (MLR‐352) 3 days before bacterial inoculations to let plants adapt to the new environment of 24°C/20°C temperature and 16/8 h light/dark cycles. All bacterial inoculants were prepared as previously described. Plant inoculations were performed using the same density of *R. solanacearum* as in the greenhouse experiments (OD_600_ = 0.7; and 5 mL) while the four potential biocontrol candidate strains were inoculated at a density of OD_600_ = 0.35 (5 mL). Three‐week‐old plants were inoculated with one bacteria (5 mL of one bacterium) or both bacteria using 5 mL of each. Negative controls included plants grown without inoculated microbes and all treatments were replicated eight times.

**TABLE 3:** Pairwise adonis test comparing the bacterial community composition between one‐ and two‐phage treatments with the bacteria‐only and negative control samples.

	F.Model	*R* ^2^	*p*‐Value	*p*‐adjusted
1‐phage vs. 2‐phages	18.8	0.14	0.001	0.006
1‐phage vs. Negative_control	12.36	0.18	0.001	0.006
1‐phage vs. Rs.UW551	14.16	0.2	0.001	0.006
2‐phages vs. Negative_control	4.53	0.05	0.013	0.024
2‐phages vs. Rs.UW551	4.44	0.05	0.012	0.024
Negative_control vs. Rs.UW551	10.49	0.32	0.001	0.006

### Analysing the importance of phages and resident bacterial taxa for disease development using partial least squares path modelling

To understand the interactions between phages, resident rhizosphere bacterial communities and *R. solanacearum* abundances on disease development, we used a partial least squares path modelling (PLS‐PM) using the R package plspm. The cross‐loading between latent variables as well as the unidimensionality in the latent variables was tested during model construction. The final model was created then by assessing ‘Phages’ (reflective indicator included as the log (PFU/mL)), ‘Microbiome’ (reflective indicator included as NMDS1 which divided the samples into shifted and centred microbiomes), ‘*Ralstonia*’ (reflective indicator included as the pathogen abundance) and ‘Disease’ (reflective indicator included as two different variables: Disease index at 21 dpi and area under the diseased curve). To compare if there were differences between shifted and centred microbiome samples, the function plspm.groups was used with the ‘bootstrap’ method with 500 repetitions.

### Statistical analysis

To assess mean differences between treatment groups, ANOVA or Kruskal–Wallis tests (based on the normality of the data) were used followed by Tukey's HSD test or Dunn's post hoc tests, respectively (package rstatix and FSA in R), and Bonferroni tests were used to adjust for multiple comparisons. The *t*_test function in rstatix package was used to compare only two groups. Pearson correlation was used to assess correlation between continuous variables. To measure the area under the disease progression curve or GFP fluorescence, we used the AUC function from the package pROC in R.

We analysed the diversity of rhizosphere bacterial communities estimated as the number of ASVs, ASVs richness, Chao1 and Shannon diversity index (at ASV level) using the vegan package (Community Ecology Package [R package vegan version 2.6–4], 2022). Beta diversity was assessed with a non‐metric multidimensional scaling (NMDS) using the metaMDS function in the R vegan package which uses Bray–Curtis distances. NMDS1 values were used to divide the samples into ‘shifted’ (NMDS1 > 0) and ‘centred’ (NMDS1 < 0) groups. To check pairwise differences, we used pairwise Permutational Multivariate Analysis of Variance Using Distance Matrices (adonis) using the pairwise.adonis function from the package pairwiseAdonis in R based on Bray–Curtis distances with 999 permutations and *p*‐values adjusted using Holm's method. All R analyses were conducted with version 4.3.2.

## RESULTS

### Phages varied in their ability to control *R. solanacearum* in vitro

We found that all four phages could produce clear inhibition halos on *R. solanacearum* lawns grown on agar plates (data not shown). Similarly, all four phages reduced *R. solanacearum* densities in liquid media compared to the control treatments without phages (Figure S2A). However, different phages had different inhibitory effects (KW_4, *N* = 20_ = 16.0, *p* = 0.03, Figure S2B): Phage PY059 showed the weakest pathogen density reduction, phage PY065 was the most effective and phages PY04 and PY045 showed intermediate efficiencies (Figure S2). These results suggest that all phages had biocontrol ability in terms of pathogen density reduction in laboratory cultures in vitro.

### One‐ and two‐phage treatments were equally effective, and phage efficiency varied between and within phage treatments

**FIGURE 1:**
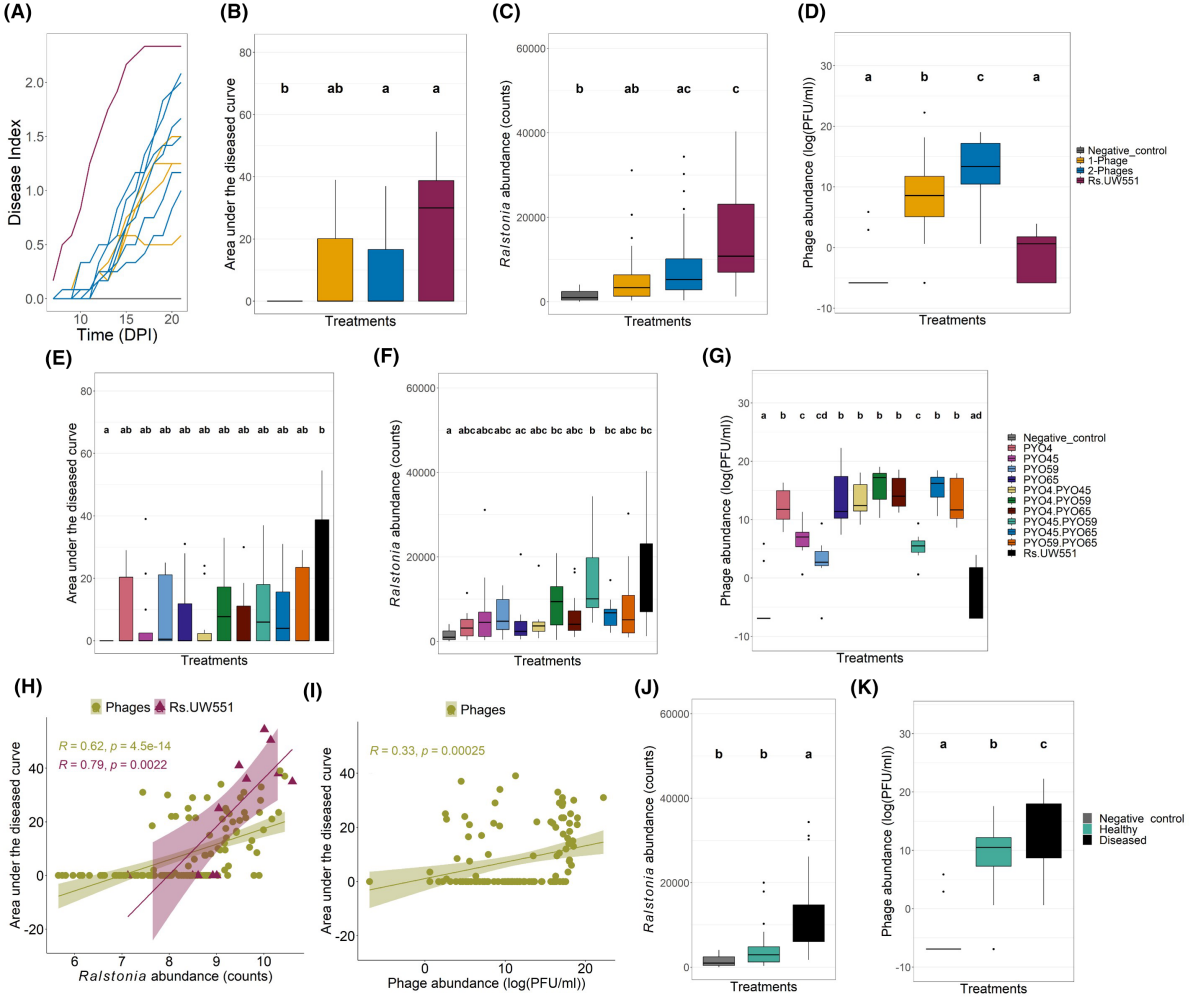
Testing phage biocontrol efficiency in a greenhouse experiment. (A) Bacterial wilt disease development over time (days post‐inoculation (DPI)). (B) Area under the diseased progression curve showing one‐phage (orange) and two‐phage (blue) treatments, positive control with the *R. solanacearum* only (purple) and negative control (grey) plants (same colour coding also used in panels C and D). (C) Pathogen relative abundances in the rhizosphere averaged over one‐ and two‐phage treatments relative to control treatments. (D) Phage abundances (log(PFU/mL)) in the rhizosphere averaged over one‐ and two‐phage treatments relative to control treatments. (E) Bacterial wilt disease development over time in terms of area under the diseased progression curve for all 10 phage treatments (colours), positive control with the *R. solanacearum* UW551 only (black) and negative control (grey; *N* = 12 plants per treatment). (F) Pathogen relative abundances in the rhizosphere for all phage and control treatments. (G) Phage abundances (log(PFU/mL)) in the rhizosphere for all phage and control treatments. (H) Pearson correlation between relative *Ralstonia* abundances (log of the counts) and the area under the disease progression curve for the mean of phage and control treatments. (I) Pearson correlation between phage abundances (log(PFU/mL)) and the area under the disease progression curve for the mean of phage treatments. (J) Pathogen relative abundances in the rhizosphere between healthy and diseased plants. (K) Phage abundances (log(PFU/mL)) in the rhizosphere between healthy and diseased plants.

To validate phage efficiency in planta, we compared the biocontrol efficacy of all phages individually and in pairwise combinations using tomato as a model plant host and UW551 *R. solanacearum* strain as the pathogen (Figure 1). Bacterial wilt disease symptoms appeared from 6 dpi in the pathogen‐only treatment (Table S9, Figure 1A), and disease symptoms were clearly delayed in the presence of phages (KW_11, *N* = 2160_ = 284, *p* = 1.76E−54; Figure 1B). We also observed that the area under the disease progression curve was lower in two‐phage compared to one‐phage treatments (KW_3, *N* = 144_ = 14.3, *p* = 0.0025, Figure 1B). However, the proportion of diseased plants in two‐phage treatments was significantly higher than one‐phage treatments at the end of the experiment (X‐squared = 35.186, *df* = 1, *p* = 1.498e−09; 33 of 72 (45.8%) vs. 15 of 48 (31.2%), respectively). Of the one‐phage treatments, PY045 was the most efficient phage even though this phage was not the most efficient at controlling *R. solanacearum* densities in vitro (Figure 1E). Of the two‐phage treatments, the most effective combination was the PY04 + PY045 treatment (Figure 1E), and these phages were also highly effective when applied as one‐phage treatments. In contrast, some combinations that consisted of highly efficient single phages (e.g. PY045 + PY065), failed to effectively control the disease (AUC compared with PY045 (*t*(351) = −2.32, *p* = 0.02 and with PY065: *t*(349) = 0.416, *p* = ns; Figure 1E)). Also, some other phage combinations (PY04 + PY059, PY045 + PY059, PY059 + PY065) failed to control the disease, potentially due to the low efficiency of PY059, which was included in all these combinations (Figure 1E). Overall, these results suggest that similar phage biocontrol efficiencies were attained when using phages in 1‐ and 2‐phage combinations, while variability in phage efficiency was large between and within phage treatments, indicative of strong phage identity effects and potential stochastic variation.

We next compared if *R. solanacearum* (read counts) and phage (log (PFU/mL)) densities could predict successful phage biocontrol outcomes based on the last time point of the greenhouse experiment (see methods). We found that *R. solanacearum* densities correlated positively with disease severity in pathogen‐only (*R* = 0.79, *p* < 0.001) and phage treatments (both 1‐ and 2‐phage treatments combined (*R* = 0.62, *p* < 0.001)) (Figure 1H). Moreover, pathogen densities were significantly reduced in all phage treatments (KW_3, *N* = 142_ = 30.4, *p* = 1.14E−6; Figure 1C,F), and when analysing phage‐treated plants only, *R. solanacearum* had higher densities in diseased plants when compared to healthy plants (KW_3, *N* = 142_ = 57.6, *p* = 3.15E−13; Figure 1J). Pathogen densities were also higher in 2‐phage compared to 1‐phage treatments (*t*(112) = −2.44, *p* = 0.0162; KW_3, *N* = 142_ = 30.4, *p* = 1.14E−6; Figure 1C), and specifically PY04 + PY059 and PY045 + PY059 (Figure 1F) treatments showed comparable pathogen densities to the pathogen‐only control treatment.

Overall, phage densities increased with the severity of disease symptoms (*R* = 0.33, *p* < 0.001; Figure 1I). Relatively higher phage densities were recovered from two‐phage compared to one‐phage treatments (*t*_(80.6)_ = −4.3, *p* < 0.001; or considering all groups *F*_3, 138_ = 60.92, *p* < 2E−16; Figure 1D) and only one of the phage combinations (PY045 + PY059) showed equally low phage densities to one‐phage treatments (Figure 1G). In general, phage densities were higher in phage‐treated diseased plants when compared to healthy plants (*F*_2, 130_ = 61.84, *p* = <2.0E−16; Figure 1K). No correlation between pathogen and phage densities was observed in one‐phage treatments. However, a negative correlation was observed in two‐phage treatments, where the reduction in *R. solanacearum* densities (log of the counts) was associated with an increase in phage densities (log (PFU/mL)) in diseased plants (*R* = −0.38, *p* = 0.03; Figure S3). These results suggest that while *R. solanacearum* densities predicted disease progression well during the greenhouse experiment, phage densities were less clearly linked with phage biocontrol efficiency. Moreover, while both one‐ or two‐phage treatments worked equally well, high variability in phage efficiency was observed within all phage treatments.

### Phage application shifts the rhizosphere microbiome diversity and composition in a subset of phage treatment replicates

**FIGURE 2:**
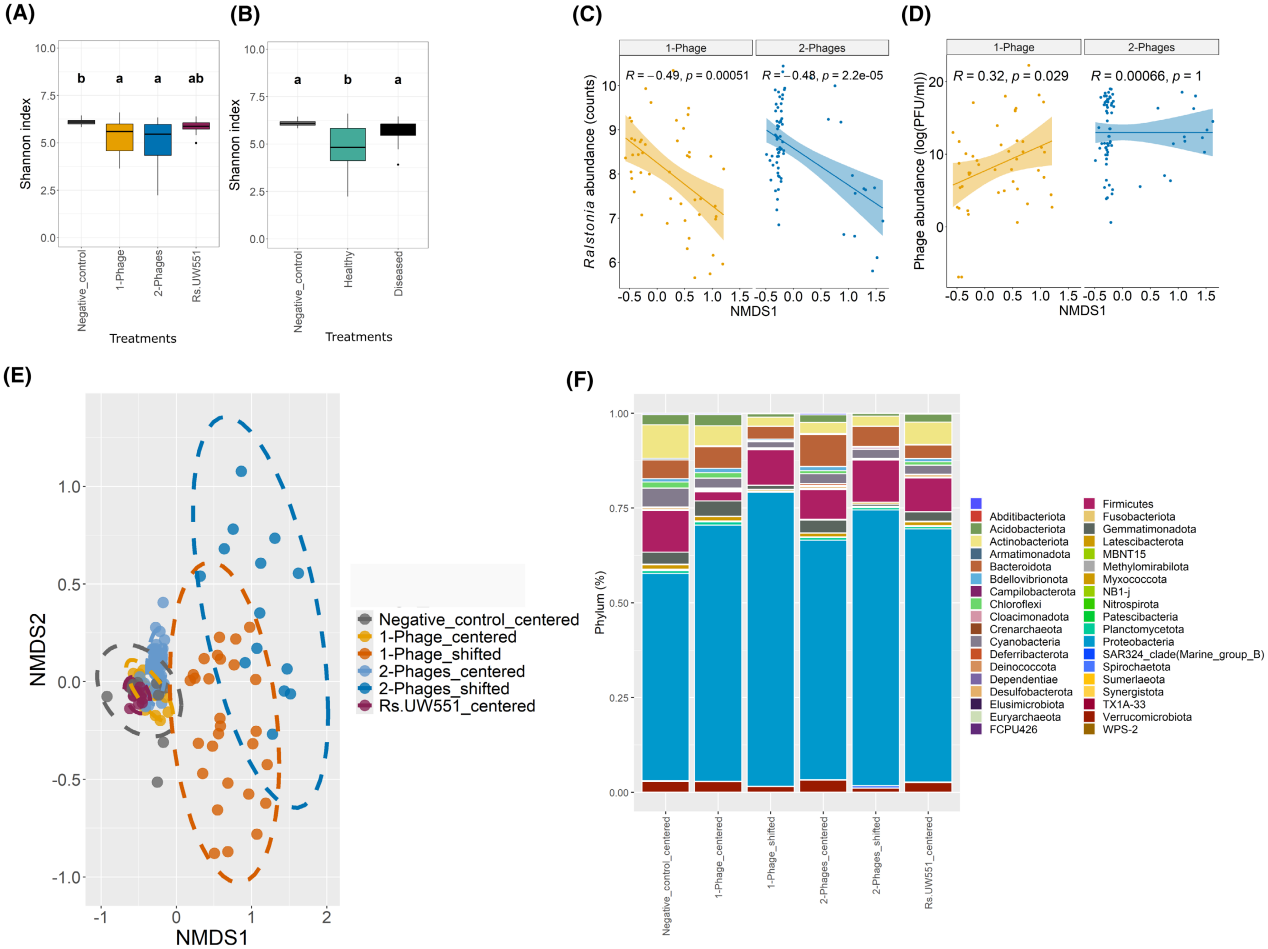
Phage application shifts bacterial community composition and relative taxa abundances in a subset of phage treatment replicates. Panels (A and B) show bacterial alpha diversities in terms of Shannon index for control and one‐ and two‐phage treatments (A) and healthy and diseased plants (B). (C, D) show correlations between *R. solanacearum* (log counts) (C) and phage (log(PFU/mL)) (D) densities along with Non‐metric multidimensional scaling (NMDS) axis 1 scores. (E) NMDS plot comparing differences in bacterial community composition in terms of beta diversity between one‐ and two‐phage treatments and control plants for ‘centred’ and ‘shifted’ plant replicates. (F) Relative bacterial phyla abundances in ‘shifted’ and ‘centred’ plant microbiome replicate samples for control and one‐ and two‐phage treatments.

**FIGURE 3:**
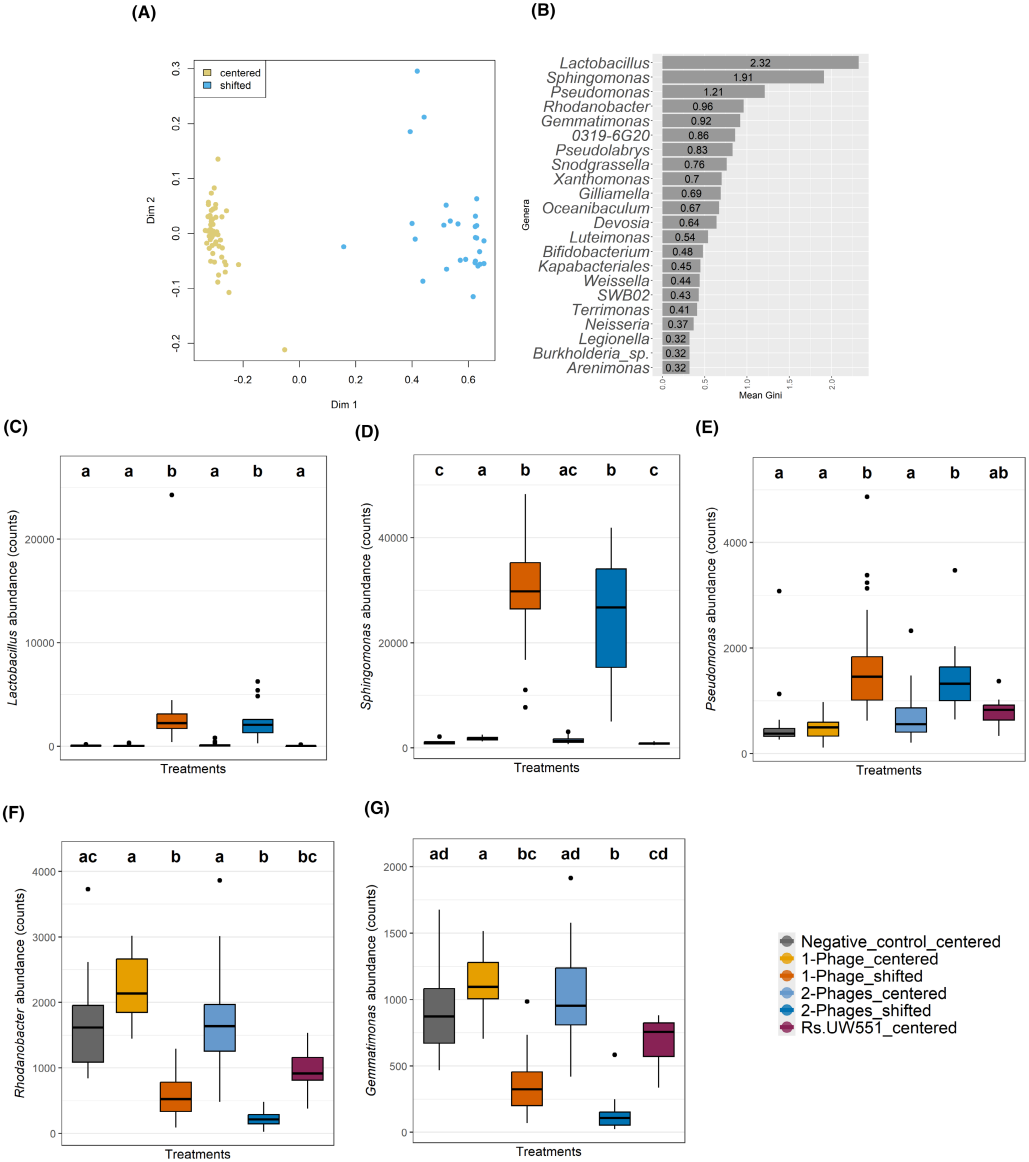
Identification of key bacterial taxa associated with shifted plant rhizosphere microbiome plant replicates. (A) Multidimensional scaling plot showing the distribution of shifted and centred rhizosphere microbiome replicate samples based on the random forest analysis. (B) The relative importance of different bacterial genera for grouping samples to ‘shifted’ and ‘centred’ microbiomes based on Gini values (in descending order). (C–G) Comparison of relative abundances of five bacterial taxa with high Gini values between control and ‘shifted’ and ‘centred’ phage treatment replicate samples (based on read counts).

To better understand the variation underlying phage efficiency within phage treatments, we focused on comparing changes in the bacterial microbiome composition at the end of the greenhouse experiment at the level of individual plants (total of 154 sequenced rhizosphere microbiome samples). As the same standardised and well‐homogenised compost mix was used for all treatment replicates, potential changes could only arise due to treatment effects or stochastic chance events. No significant differences in the bacterial alpha diversity (Shannon index) were found between one‐ and two‐phage treatments, while both phage treatments had significantly lower diversities compared to the negative control treatment (KW_3, *N* = 142_ = 18.1, *p* = 4.17E−4; Figure 2A, Table S10). Moreover, bacterial diversity was lower in the healthy plants when compared to diseased plants (*F*_2, 130_ = 22.88, *p* = <2.6E−09, Figure 2B). Significant differences were also found in terms of bacterial community composition assessed by NMDS analysis (stress = 0.11; Figure 2E) and pairwise Adonis test confirmed that one‐ and two‐phage treatments differed significantly from each other (*R* ^2^ = 0.14, *p*‐adj = 0.006), from the negative control (1‐phage: *R* ^2^ = 0.18, *p*‐adj = 0.006; 2‐phage: *R* ^2^ = 0.05, *p*‐adj = 0.024) and pathogen‐only treatment (1‐phage: *R* ^2^ = 0.2, *p*‐adj = 0.006; 2‐phage: *R* ^2^ = 0.05, *p*‐adj = 0.024) (Table 3). Crucially, while all control microbiome samples clustered tightly together, some one‐ and two‐phage plant replicate samples showed a shift in their community composition along both NMDS1 and NMDS2 axes (Figure 2E). Interestingly, this shift in microbiome composition could be associated with a reduction in *R. solanacearum* densities along with increasing values on NDMS1 for both one‐phage (*R* = 0.49, *p* = 5.1E−03; Figure 2C) and two‐phage treatment replicate samples (*R* = 0.48, *p* = 2.2E−05; Figure 2C). Moreover, movement along the NDMS1 was positively correlated with higher phage abundances, especially in one‐phage treatment replicate samples (*R* = 0.32, *p* = 0.029; Figure 2D). Negative values on NMDS2 correlated with a reduction in *R. solanacearum* densities, but only in one‐phage treatments (*R* = 0.46, *p* = 0.001, Figure S4, Figure 3A). For further analyses, we classified our microbiome samples using the NMDS1 axis where samples with values >0 were considered as ‘shifted’ microbiome replicate samples, and samples with values <0 were considered as ‘centred’ microbiome replicate samples.

Interestingly, while the shift in the microbiomes of a subset of phage treatment replicates was associated with reduced pathogen and increased phage densities, these replicate samples were not enriched in the healthy plant category (Figure S5A). However, some phage treatments were underrepresented in the shifted microbiome replicate subset (Figure S5B). For example, a significant underrepresentation of plants treated only with PY059 (*χ* ^2^ = 4.3363, *df* = 1, *p*‐value = 0.01865) or with PY04 + PY045, PY04 + PY059, PY045 + PY059 phage combinations was observed in the shifted plant replicate subset (*χ* ^2^_(1, *N* = 57)_ = 3.6, *p* = 0.03; *χ* ^2^_(1, *N* = 57)_ = 4.76, *p* = 0.01; *χ* ^2^_(1, *N* = 57)_ = 4.76, *p* = 0.01, respectively). Moreover, no plants treated with PY045 + PY065 or PY059 + PY065 phage combinations were included in the shifted microbiome samples.

To assess if certain bacterial taxa were enriched or reduced in the shifted microbiome replicate samples, we performed a random forest analysis using all 118 microbiome samples covering all phage treatment plant replicates (no negative or positive control samples). We randomly divided the data set into training (*N* = 80) and test (*N* = 38) data sets. Based on 1000 trees per data set, the first random forest model that differentiated taxa between shifted and centred microbiomes showed an out‐of‐bag (OOB) error rate of 0% and 0% classifying error in each of the categories, confirming the high robustness of the model (Figure 3B). The importance of each classifying taxa was explored at genera level using Gini values >0.3. The genera with the highest values were *Lactobacillus*, *Sphingomonas*, *Pseudomonas* and *Rhodanobacter* (Figure 3C–F, Figure S6, Table S11). Of the five most important taxa, *Lactobacillus*, *Sphingomonas and Pseudomonas* were significantly enriched and *Rhodanobacter* and *Gemmatimonas* significantly reduced in shifted microbiome replicate samples in both one‐phage and two‐phage treatments. Similarly, other taxa with lower Average Mean Gini values, such as *Snodgrassella*, *Xanthomonas*, *Gilliamella*, *Oceanibaculum*, *Bifidobacterium*, *Weissella* and *Neisseria*, were enriched in the shifted microbiome samples, while 0319‐6G20, *Pseudolabrys*, *Devosia*, *Luteimonas*, *Kapabacteriales*, SWB02, *Terrimonas*, *Legionella*, *Burkholderia*‐*Caballeronia*‐*Paraburkholderia* and *Arenimonas* were reduced in the shifted microbiome samples (Figure S6). Together, these results suggest that phages drove shifts in the tomato rhizosphere microbiome composition in a subset of phage treatment replicates, which were also associated with reduced *R. solanacearum* and increased phage densities.

### Enriched bacterial taxa in shifted microbiomes can biocontrol *R. solanacearum* in vitro and in planta

**FIGURE 4:**
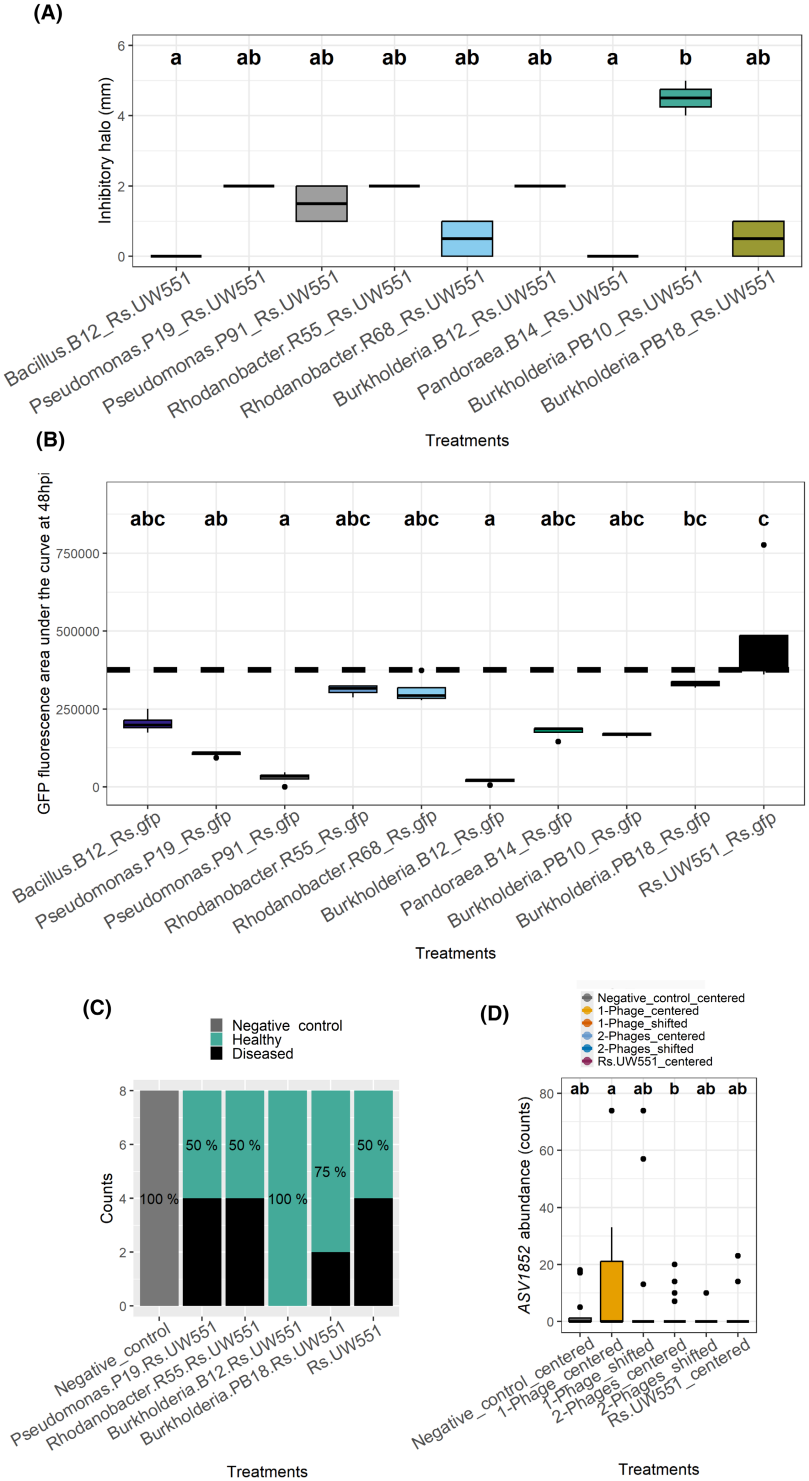
Direct experiments testing the in vitro and in planta biocontrol effect of selected rhizosphere taxa. (A) Inhibitory potential of the nine selected bacteria against *R. solanacearum* based on inhibition halos (mm) observed in spotting assays. (B) Area under the growth curve during 48 h in liquid culture measuring GFP (emission at 509 nm) when growing individually or when growing with GFP‐tagged *R. solanacearum*. (C) Number of healthy and diseased plants in plants treated *Pseudomonas* P19, *Rhodanobacter* R55, *Burkholderia* B12 and *Burkholderia* PB18 and *R. solanacearum*. (D) Comparison of relative abundance of ASV1852, showing the highest similarity to *Burkholderia* B12 between control and ‘shifted’ and ‘centred’ phage treatment samples (based on read counts).

To test if the reduction in *R. solanacearum* densities within shifted microbiome replicates could be explained by enrichment of potentially pathogen‐suppressing taxa, we isolated >100 bacterial colonies at the end of the greenhouse experiment. We were able to isolate the following bacteria with high Gini values: four isolates of *Burkholderia‐Caballeronia‐Paraburkholderia* sp., one *Bacillus* sp. isolate, two *Pseudomonas* sp. and two *Rhodanobacter* sp. isolates that were likely fast growers in the isolation media used. Using in vitro lab experiments, we found that both *Pseudomonas* (strains P19 and P91), one *Rhodanobacter* (R55) and the three *Burkholderia* sp. (B12, PB10 and PB18) (Figure 4A, Table 2) showed clear inhibition halos when grown over a lawn of *R. solanacearum* strain. Similar inhibitory effects were found in co‐cultures grown in liquid media over a 48‐h time course (KW_9, *N* = 40_ = 37.0, *p* = 2.6E−5), and this inhibition was especially prominent with both *Pseudomonas* strains and the *Burkholderia* B12 strain (Figure 4B). For the in planta disease suppression greenhouse assays, we selected *Pseudomonas* P19 and *Burkholderia* B12 isolates as potential biocontrol strains and also included *Rhodanobacter* R55 and *Burkholderia* PB18 as potential ‘negative control’ strains with expected low biocontrol potential based on our in vitro assays. We found that the *Burkholderia* B12 strain was able to completely inhibit bacterial wilt symptoms (Figure 4C), while *Burkholderia* PB18 strain was also quite effective, with only two of eight plants showing disease symptoms. In contrast, *Pseudomonas* P19 and *Rhodanobacter* R55 were unable to delay or reduce disease development. Due to the high biocontrol activity of *Burkholderia* B12, we compared the 16S sequence obtained by Sanger of this strain, with the metabarcoding data, to identify the ASV1852 corresponding with the highest similarity (99.6% similarity, *E*‐value = 5.53E−130) and observed that the abundance of this taxon was significantly increased in the centred 1‐phage replicate samples, corresponding with the abundance profile seen for the whole genera (Figure S5). These results demonstrate that changes in the composition of bacterial taxa in phage treatment microbiomes were also associated with functional changes in the suppressiveness of these communities.

### Partial least squares path modelling (PLS‐PM) suggests synergy between phages and the resident rhizosphere microbiota

**FIGURE 5:**
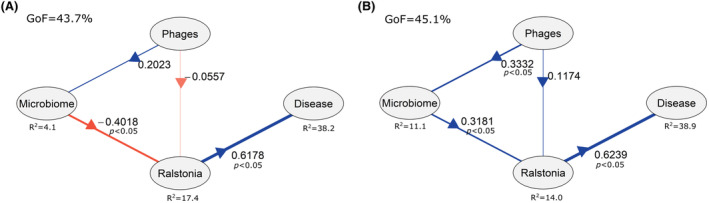
Partial least squares path models (PLS‐PM) comparing significant associations between different variables on pathogen densities and bacterial wilt disease progression. (A) Path coefficients for the PLS‐PM in the shifted microbiome sample replicates. (B) Path coefficients for the PLS‐PM in the centred microbiome sample replicates. In both models, ‘Phages’ refer to phage densities (log(PFU/mL)), ‘Microbiome’ to NMDS1 score, ‘*Ralstonia*’ to pathogen densities (read counts) and ‘Disease’ to disease score at 21 dpi and the area under the disease progression curve. Positive and negative associations between variables are shown in blue and red, respectively, and the arrows show the direction of effects. The *R* ^2^ values show the percentage of variation explained by the models.

To explore direct and indirect relationships between different variables associated with bacterial wilt disease severity, a partial least squares path modelling (PLS‐PM) was used. Specifically, we explored the effects of phages and the shift in the microbiome composition on *R. solanacearum* densities and disease severity separately within the centred (NMDS1 < 0) and the shifted (NMDS1 > 0) microbiome groups, resulting in two separate PLS‐PM models (Figure 5). The goodness of fit (GoF) for the shifted microbiome model reached 43.7%. As expected, we observed a significant positive effect of ‘*Ralstonia*’ on ‘Disease’ (0.62), indicating that pathogen abundances correlated positively with the disease severity. Moreover, the microbiome composition, reflected by NMDS1 value, had a negative effect on ‘*Ralstonia*’ (−0.4), indicative of the potential suppressive effect of microbiota on the pathogen. The ‘Phages’ had no significant effects on ‘Microbiome’ or ‘*Ralstonia*’ in this model, suggesting that phage densities were not clearly linked with disease progression. The goodness of fit (GoF) for the centred microbiome model was similar to the shifted microbiome model (GoF = 45.1%). However, a contrasting effect of the ‘Microbiome’ on ‘R*alstonia*’ was found; microbiomes showed a positive association with pathogen densities (0.32), suggesting that some rhizobacteria may have facilitated disease progression. A bootstrap test with 500 iterations was conducted to confirm the contrasting effects of ‘Microbiome’ on ‘*Ralstonia*’ between the shifted and centred microbiome models (*t*(116) = 4.96, *p* < 0.001). Moreover, ‘Phages’ were positively associated with ‘Microbiome’ (0.33) in the centred model, suggesting that phages had indirect effects on the rhizosphere microbiome composition in this sample group. Together, these models suggest that resident microbiome composition was an important factor contributing to the abundance of the pathogen and severity of disease incidence, explaining the variation in phage biocontrol efficiency within phage treatment replicates.

## DISCUSSION

In this study, we compared how phage identities and their application either alone or in two‐phage combinations affected the efficiency of phage biocontrol against *R. solanacearum* phytopathogenic bacterium. We found that the species identity of applied phages and their combinations was more important in explaining phage efficiency compared to using phages either alone or as two‐phage combinations. Moreover, bacterial wilt disease biocontrol was better explained by a reduction in pathogen densities instead of corresponding increases in phage densities. Despite improved disease biocontrol, large between‐replicate variation was observed in both one‐ and two‐phage treatments, which was stochastic within treatments but was associated with non‐random changes in rhizosphere microbiome composition across all phage treatments, with certain taxa showing consistent enrichment or reduction in their relative abundances. While these microbiome shifts took place equally often in diseased and healthy plants, they were associated with a reduction in pathogen and an increase in phage densities, indicative of improved phage biocontrol. While our results are in line with previous research, which demonstrated that phage biocontrol applications can lead to taxonomic and functional changes in the resident rhizosphere microbiota (Wang et al., 2019, 2024), we further show that such effects can be stochastic and arise quickly between initially identical plant replicates during one plant growth cycle.

Interestingly, we found that both 1‐ and 2‐phage treatments reduced bacterial wilt disease incidence and pathogen densities to a similar degree. Overall, a reduction in *R. solanacearum* densities predicted bacterial wilt disease incidence well in both 1‐ and 2‐phage treatments. However, in contrast to previous studies (Wang et al., 2019, 2024; Yang et al., 2023), phage densities predicted bacterial wilt disease incidence less well in our data, even though phages increased in their density in the presence of *R. solanacearum* during the experiment. A previous study showed that increasing the number of phages during phage biocontrol can lead to improved bacterial wilt disease control (Wang et al., 2019), where the best results were obtained when four phages were applied simultaneously. However, no significant benefits of using two‐phage combinations were observed in this study (Wang et al., 2019), which also aligns well with other studies reporting that phage cocktails do not always improve phage biocontrol efficiency relative to the application of single phages (Álvarez et al., 2019; Rabiey et al., 2020). Instead, previous results suggested that at least three phages might be needed to efficiently control *R. solanacearum* in the rhizosphere (Álvarez et al., 2019; Wang et al., 2019). Our results further suggest that the identity of phages and phage combinations had a big effect on their efficiency. While two of the phages used in this study (PY04 and PY065) were genetically highly similar, the other two phages (PY045 and PY059) were completely dissimilar. Interestingly, we observed that using dissimilar phages together in combination, such as PY04 + PY059, PY045 + PY059 and PY059 + PY065, led to poorer biocontrol outcomes in terms of higher *R. solanacearum* abundances and increased disease incidence. However, on some occasions, combining dissimilar phages, such as PY04 + PY045, led to very good biocontrol outcomes, which suggests that phage genetic dissimilarity alone is a poor predictor of combination efficiency. In line with these results, it was previously found that combining even highly genetically similar phages (>99%) can lead to significant improvement in phage biocontrol efficiency against *R. solanacearum* (Wang et al., 2019). Importantly, our results suggest that combining phages can reduce phage biocontrol efficiency relative to applying phages alone. This could be explained by the transition to persistent cells which help bacteria survive the phage stress by reducing their metabolic activity (Fernández‐García et al., 2024), phage‐resistant phenotypes arising by genetic mutation (Wang, Wang, et al., 2023) or more likely phage–phage competition from the same host cells (Refardt, 2011). Moreover, the reduced efficiency of some two‐phage treatments might have been due to the poor efficiency of phage PY059, which was observed to lack the HNH gene (Text S1), which is required for efficient phage packaging during production of virions (Kala et al., 2014). Together, these results suggest that using more phages during phytopathogen biocontrol may not always result in improved disease suppression, and that combining phages based on their genetic dissimilarity might be a bad proxy for improving their efficiency.

In addition to significant between‐phage treatment variation, we observed a large intra‐treatment variation in phage efficiency within all one‐ and two‐phage treatments. Interestingly, while a subset of phage treatment replicates showed a clear shift in their microbiome composition, these shifts were not consistently associated with healthy or diseased plants. However, they were only observed in phage treatments and were correlated with a reduction in pathogen and an increase in phage densities, indicative of improved phage biocontrol. Crucially, the shifted microbiomes were consistently associated with the enrichment or reduction of certain bacterial genera, including Sphingomonadaceae and Pseudomonadaceae, which have previously been associated with the suppressiveness of rhizosphere microbiome of the resistant ‘Hawaii 7996’ tomato cultivar against *R. solanacearum* (Kwak et al., 2018). We were able to isolate candidate bacterial taxa from our end‐point samples of the greenhouse experiment to test their disease suppressiveness against *R. solanacearum* in vitro and in planta experiments. While some of these strains suppressed *R. solanacearum* consistently both in vitro and in planta, some strains that worked well in vitro failed to control disease in planta. Despite several attempts, we failed to isolate *Sphingomonas* from our end‐point samples even though it was highly enriched based on sequence data. Overall, isolates classified as *Burkholderia* were the most effective at controlling *R. solanacearum* in planta validation experiments. Previous studies have shown that *Burkholderia* can be enriched in the rhizosphere in response to plant stimuli (Luo et al., 2021) and when controlling *R. solanacearum* with integrated biocontrol approaches (Hu et al., 2021). Members of *Burkholderia* can suppress various soil‐borne pathogens (Eberl & Vandamme, 2016) and could hence be an important taxon for soil suppressiveness to bacterial wilt. Interestingly, the most suppressive *Burkholderia* species we tested was enriched in centred microbiome samples in one‐phage treatments, suggesting that phages likely affected the suppressiveness of rhizosphere microbiota also in non‐shifted rhizosphere bacterial communities. A few previous studies have also found that the application of pathogen‐specific phages can lead to indirect changes in the microbiome composition (Wang et al., 2024) and enrichment of disease‐suppressive taxa belonging to *Streptomyces* and *Nocardioides* (Wang et al., 2024). As the applied phages were specific to *R. solanacearum*, the likely mechanism for the enrichment was the release of niche space by the phages that efficiently reduced the relative pathogen densities in the rhizosphere. In support of this, a recent study showed that changes in the resident microbiome taxa composition were clearer when phages could reduce the relative abundances of *R. solanacearum* more efficiently, which was also positively correlated with increased phage densities (Wang et al., 2024). Our results align well with this previous study as the shifts in the microbiome composition were more often observed when the reduction in the *R. solanacearum* relative abundances was clearer. However, the enriched taxa were different, which is not surprising as the compost substrate and associated resident bacterial communities were different between these experiments. Our findings also suggest that even though certain taxa responded consistently to phage application, in the shifted bacterial communities, composition in both 1‐ and 2‐phage treatments diverged, while centred communities remained very similar to each other. As several bacterial taxa, including *Bacillus*, *Pseudomonas* and *Streptomyces* have been linked with bacterial wilt disease‐suppressive soils in previous studies (Wang et al., 2017, 2024; Wei et al., 2019; Yang et al., 2023), it is possible that any competitive taxa present in the rhizosphere microbiome might be able to respond and increase in relative abundance in response to phage infection‐mediated release of the niche space.

The shift in the microbiome composition within phage treatments was likely stochastic as all the plants in the greenhouse experiment were started in identical conditions using the same batch of well‐mixed and homogenised compost substrate (John Innes No2). While the initial microbiomes were highly similar between all the plants (based on the tight clustering of negative control samples), a subset of plant replicates diverged and experienced a shift in their microbiome composition during one plant growth cycle. Crucially, the shifts in microbiome composition were not taxonomically random and the same bacterial taxa were observed to shift between different phage treatments. Even though we tried to make the starting conditions as similar as possible between the plant replicates, it is possible that some small differences in initial microbiomes were still present. Such small initial differences in microbiome composition have previously been shown to determine whether plants get infected by *R. solanacearum* or if they remain healthy during the subsequent tomato growth cycle (Wei et al., 2019). In another study, Gu et al. (2022) showed that initially homogeneous soils can quickly diverge into compositionally different microbiomes that later become associated with healthy or diseased plants. As we lack initial and temporal sampling of all plant rhizosphere microbiome replicates, we cannot rule out either of these hypotheses. However, as we used highly controlled experimental conditions as evidenced by tight clustering of negative control replicates, our findings are more likely explained by the scenario proposed by Gu et al. (2022), where initially similar microbiomes rapidly diverged between plant replicates. To confirm this in the future, time‐series data are needed.

Our findings have several important implications for phage biocontrol of phytopathogens. First, our results demonstrate that when applied through root drenching, phage ability to control *R. solanacearum* densities depends on the responses of the resident rhizosphere microbiota, and crucially, we found that this effect was stochastic among phage treatment replicates, introducing variability in phage biocontrol efficiency. This could have important repercussions for applying phages successfully in the field, where differences in the soil microbiome composition are likely to be greater (Hannula et al., 2020). Even though variability in rhizosphere microbiome composition could introduce more variability into the success of phage biocontrol, current data suggest that different resident bacterial taxa could provide disease suppression in synergy with applied phages (Wang et al., 2019, 2024). It is hence possible that different rhizosphere microbiomes contain functionally redundant bacteria that can suppress soil‐borne pathogens and be enriched in response to phage application despite belonging to different taxonomic groups. Moreover, some of the disease‐suppressive taxa we validated showed reduction in their relative abundance in response to phage application. Hence, future work should explore phage effects at the bacterial community level to better understand the net effects of microbiome changes on disease suppression. Finally, what triggered the microbiome shifts in a subset of phage treatment replicates remains inconclusive. One potential and underexplored factor could be soil viromes, which have been linked with changes in the soil suppressiveness to *R. solanacearum* via effects on the disease‐suppressive bacterial taxa (Yang et al., 2023). Further work should also assess if these results depend on the phage application method, for example, by evaluating if rhizosphere changes also take place when the phages are applied as seed coating (Erdrich et al., 2024) instead of root drenches. In conclusion, our data suggest that the presence of resident microbiome can complicate phage biocontrol outcomes in plant rhizosphere by introducing variation in treatment efficiency.

## AUTHOR CONTRIBUTIONS

**Sara Franco Ortega:** Investigation; writing – original draft; methodology; visualization; software; formal analysis; data curation. **Bryden Fields:** Investigation; methodology; software; formal analysis; data curation. **Daniel Narino Rojas:** Investigation; methodology. **Lauri Mikonranta:** Investigation; methodology. **Matthew Holmes:** Investigation; methodology. **Andrea L. Harper:** Funding acquisition; writing – review and editing; project administration; supervision; resources. **Ville‐Petri Friman:** Project administration; supervision; resources; writing – review and editing; funding acquisition; conceptualization; visualization.

## CONFLICT OF INTEREST STATEMENT

The authors declare that they have no competing interests.

## Supporting information


Appendix S1

Table S2

Table S3

Table S4

Table S5

Table S6

Table S7

Table S8

Table S9

Table S10

Table S11

## Data Availability

The raw data of the 16S are available in NCBI Sequence Read Archive (SRA) BioProject PRJNA1010659. Bacteriophage genomes raw data are deposited in SRA BioProject PRJNA1076092. Genomes with full annotations are available at GenBank PP358254–PP358257 and locally at the University of York server: https://webfiles.york.ac.uk/Harper/Phages/. All the scripts for the 16S analysis can be found at https://github.com/sfortega/Ralstonia‐phage/.
