# Supplementary material for: Phage biocontrol success of bacterial wilt depends on synergistic interactions with resident rhizosphere microbiota
Source: Microb Biotechnol. 2024 Nov 13;17(11):e70049. doi: 10.1111/1751-7915.70049 (PMC11561305; doi:10.1111/1751-7915.70049)
Supplement: Supplementary file 1 — Appendix S1 [file MBT2-17-e70049-s009.docx]

**Supplementary text**

**Phage genome assemblies**

SPAdes (v3.14.9) (Bankevich et al., 2012) was used to assemble the phage genomes with the option “only assembler”. Genome assembly coverage was determined using BBMap covstats. After that, the largest contig was extracted as complete phage genome using in-house python scripts followed by polishing with Pilon (v1.24) using the –frags option (Walker et al., 2014). Annotation was performed with Prokka (1.14.6) (Seemann, 2014) using the external PHROGs HMM database (Shen and Millard, 2021) prokka virus database and the external caudovirales database annotations to determine the gene orientations and positions and with. Programme checkV (0.9.0) was used to determine any potential termini and to identify any repeats produced as an assembly artefact on the 5’ or 3’ ends of the phage contig. Any repeat regions identified on the 5’ or 3’ contig ends were removed with an in-house python script. Bacteriophage genomes were reoriented when the *terL* gene (or *terS* gene if the *terL* gene was not identified) was found on the negative strand. Genomes were further reordered to the start of the *terL* gene (or *terS* gene if the terL gene was not identified). After genome repeat removal, reorientation and reordering, genomes contigs were polished again with Pilon to produce the final phage genome assemblies. The PY04 genome was assembled into a single contig 40,412bp in length with GC content of 58.91%. PY04 matched PHAGE_Ralsto_RsoP1EGY (NC_047946) when analysed using PHASTER (Score =123). The PY045 genome was assembled into one contig 61,247bp in length and with GC content of 64.45%, and was identified as PHAGE_Burkho_DC1 (NC_018452) using PHASTER (score =130). PY059 had the shortest genome size with a single contig of 39,534 bp and a GC content of 53.74%. This bacteriophage was identified as PHAGE_Salmon_SEN34 (NC_028699) using PHASTER (score =120). Lastly, PY065 with a genome size of single contig of 40,410bp and GC content of 58.93% was identified as PHAGE_Ralsto_RsoP1EGY (NC_047946) using PHASTER (score =126). Prokka PHROGs annotation identified 51 gene models in PY04, 71 gene models in PY045, 60 gene models in PY059 and 51 gene models in PY065.

**Orthologous genes are different between phages**

SYNIMA analysis using the peptide sequence identified 42 orthogroups between PY04 and PY065. Conversely, 91.4% and 88.3% of genes in PY045 and PY059, respectively, could not be assigned to orthogroups (Table S1, Table S2). To establish why PY059 was less effective at suppressing disease than the other phage, we explored whether any orthogroups were shared between PY04, PY045 and PY065, but missing in PY059 (Figure S1, 4th column). Two orthogroups were found to be present in the three effective phages, but not PY059; Orthogroup 1 was formed by the proteins named ANGOEMEO_00022 (PY04), DGMDFAKB_00035 (PY045), DGMDFAKB_00053 (PY045) and CGDPDFMH_00022 (PY065), whilst orthogroup 8 was formed by the proteins named ANGOEMEO_00038 (PY04), DGMDFAKB_00022 (PY045) and CGDPDFMH_00038 (PY065) (Table S4).

Swissmodel in Expasy showed that from the proteins belonging to the orthogroup 1, ANGOEMEO_00022 had 24.3% similarity with 1u3e.1.D HNH homing endonuclease I-HmuI, with DGMDFAKB_00035 DGMDFAKB_00053 showing 27% and 28.43% similarity respectively with the same model. PHASTER also showed similarity in the genome coordinates of DGMDFAKB_00035 with (23807- 24277) PHAGE_Salmon_FelixO1_NC_005282: Putative HNH endonuclease; PP_00034; phage (gi38707748) with an E-value of 4.87E-18. HNH proteins are key components of the bacteriophage DNA packaging machine (Kala et al., 2014; Quiles-Puchalt et al., 2014; Zhang et al., 2017) and are usually associated to terminases in the bacteriophage genome.

The proteins of the orthogroup 8, showed similarity with a mitochondrial ATP synthase 6yo0.1.K subunit b and 6yny.2.A subunit b (21.28% for the ANGOEMEO_00038 and 21.28% for the CGDPDFMH_00038).

**References**

Bankevich, A., Nurk, S., Antipov, D., Gurevich, A.A., Dvorkin, M., Kulikov, A.S., et al. (2012) SPAdes: A New Genome Assembly Algorithm and Its Applications to Single-Cell Sequencing. *J Comput Biol* **19**: 455.

Kala, S., Cumby, N., Sadowski, P.D., Hyder, B.Z., Kanelis, V., Davidson, A.R., and Maxwell, K.L. (2014) HNH proteins are a widespread component of phage DNA packaging machines. *Proc Natl Acad Sci USA* **111**: 6022–6027.

Quiles-Puchalt, N., Carpena, N., Alonso, J.C., Novick, R.P., Marina, A., and Penadés, J.R. (2014) Staphylococcal pathogenicity island DNA packaging system involving cos-site packaging and phage-encoded HNH endonucleases. *Proc Natl Acad Sci USA* **111**: 6016–6021.

Seemann, T. (2014) Prokka: rapid prokaryotic genome annotation. *Bioinformatics* **30**: 2068–2069.

Shen, A. and Millard, A. (2021) Phage Genome Annotation: Where to Begin and End. *Phage* **2**: 183.

Walker, B.J., Abeel, T., Shea, T., Priest, M., Abouelliel, A., Sakthikumar, S., et al. (2014) Pilon: An Integrated Tool for Comprehensive Microbial Variant Detection and Genome Assembly Improvement. *PLoS One* **9**: e112963–e112963.

Zhang, L., Xu, D., Huang, Y., Zhu, X., Rui, M., Wan, T., et al. (2017) Structural and functional characterization of deep-sea thermophilic bacteriophage GVE2 HNH endonuclease. *Sci Rep* **7**: 1–13.

**Supplementary Figures**


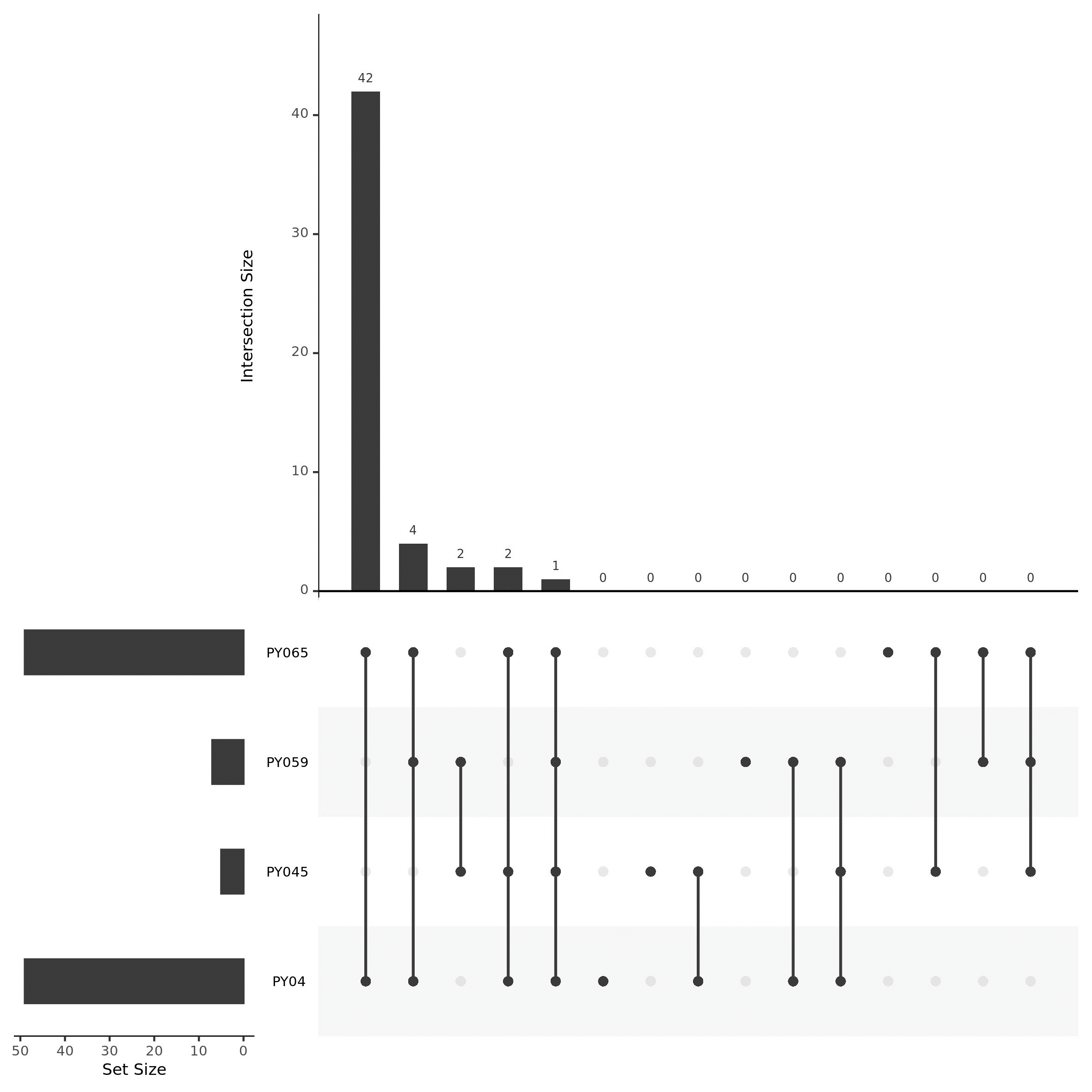


**Figure S1**. **Measuriement of genetic similarity between four phages**. Venn diagram of the shared orthogroups (based on Orthofinder results) between the four phages (PY04, PY045, PY059, PY065). The number of common orthogroups is indicated by connected bars in the intersection plot.


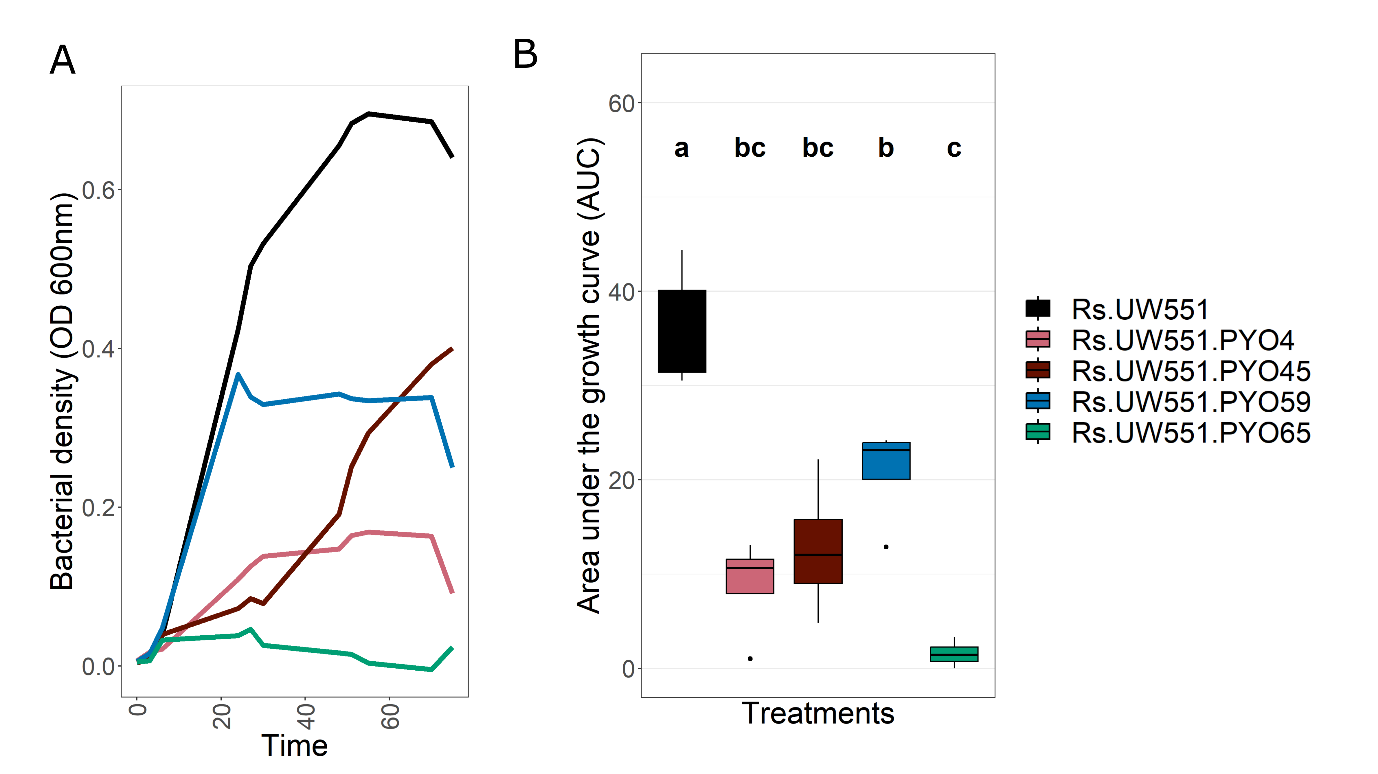


**Figure S2**. **Liquid phage assays show inhibitory effect against *R. solanacearum*. A** Growth curve during 72h of each of the four phages (PY04 (pink); PY045 (red); PY059 (blue); PY065 (green)). **B**. Area under the growth curve for *R. solanacearum* UW551 growing alone and in the presence of each of the four phages (N=4). In b) different letters above the boxplots denote significant pairwise differences between the treatments.


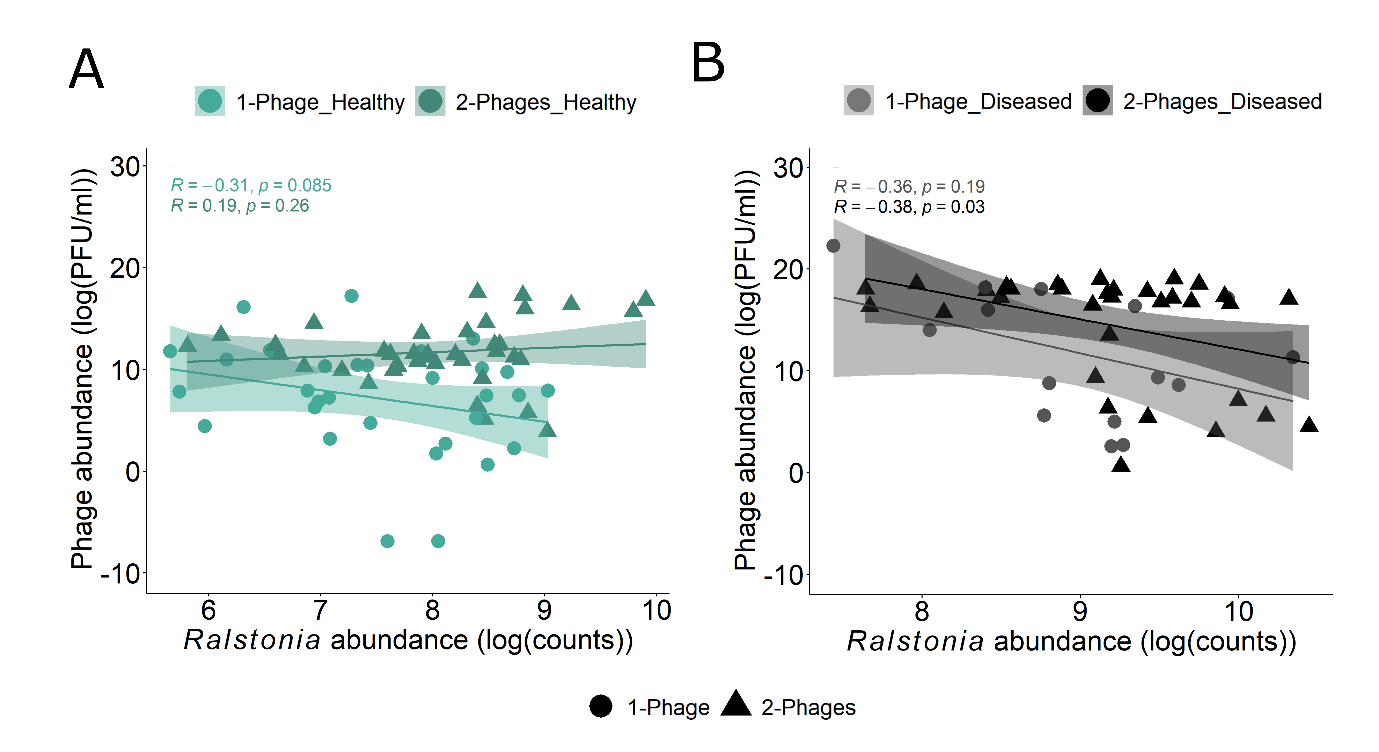


**Figure S3**. **Correlation between Ralstonia and phage abundance in 1- and 2-phage treatments for healthy and diseased plants. A**. Pearson correlation between *Ralstonia* (log of the counts) and phage abundances in 1- and 2-phage treatments in healthy plants. **B**. Pearson correlation between *Ralstonia* (log of the counts) and phage abundances in 1- and 2-phage treatment in diseased plants.


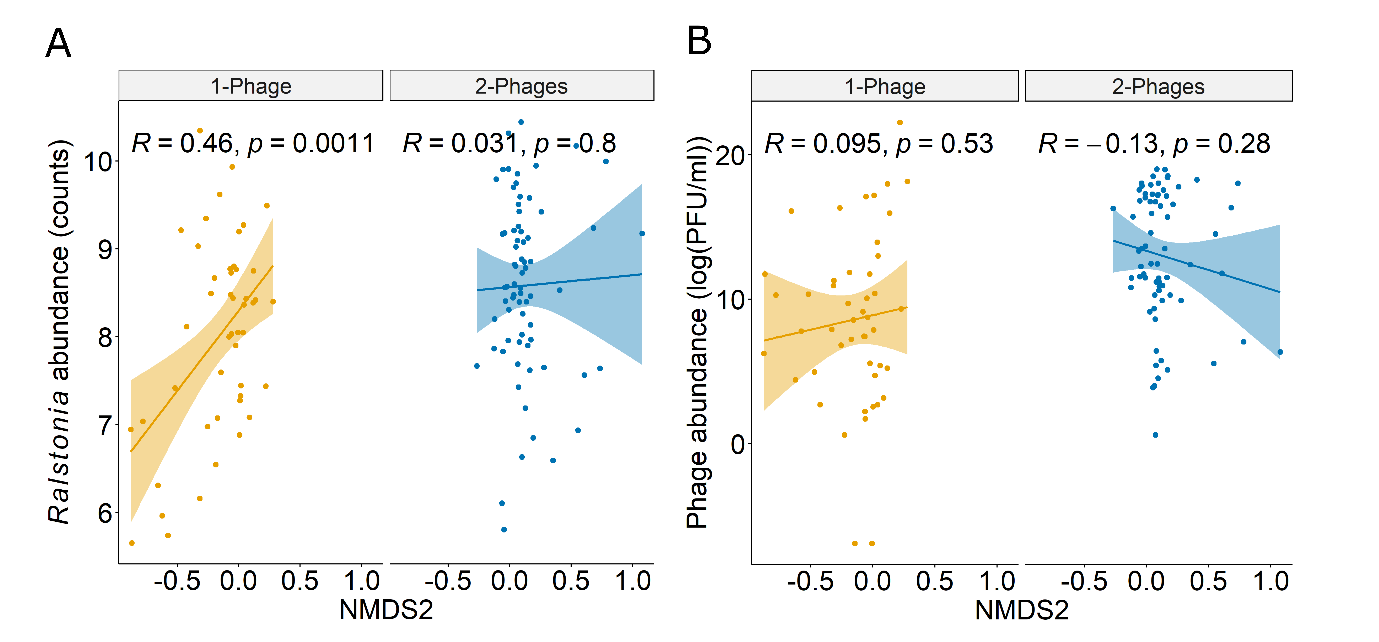


**Figure S4**. **Pathogen and phage abundance correlations with rhizosphere microbiome beta-diversity. A.** Correlations between NMDS2 axis and *Ralstonia* (log counts) abundances in 1- and 2-phage treatments. Correlation between NMDS2 axis and phage abundances (log(PFU/ml)) in 1- and 2-phage treatments.


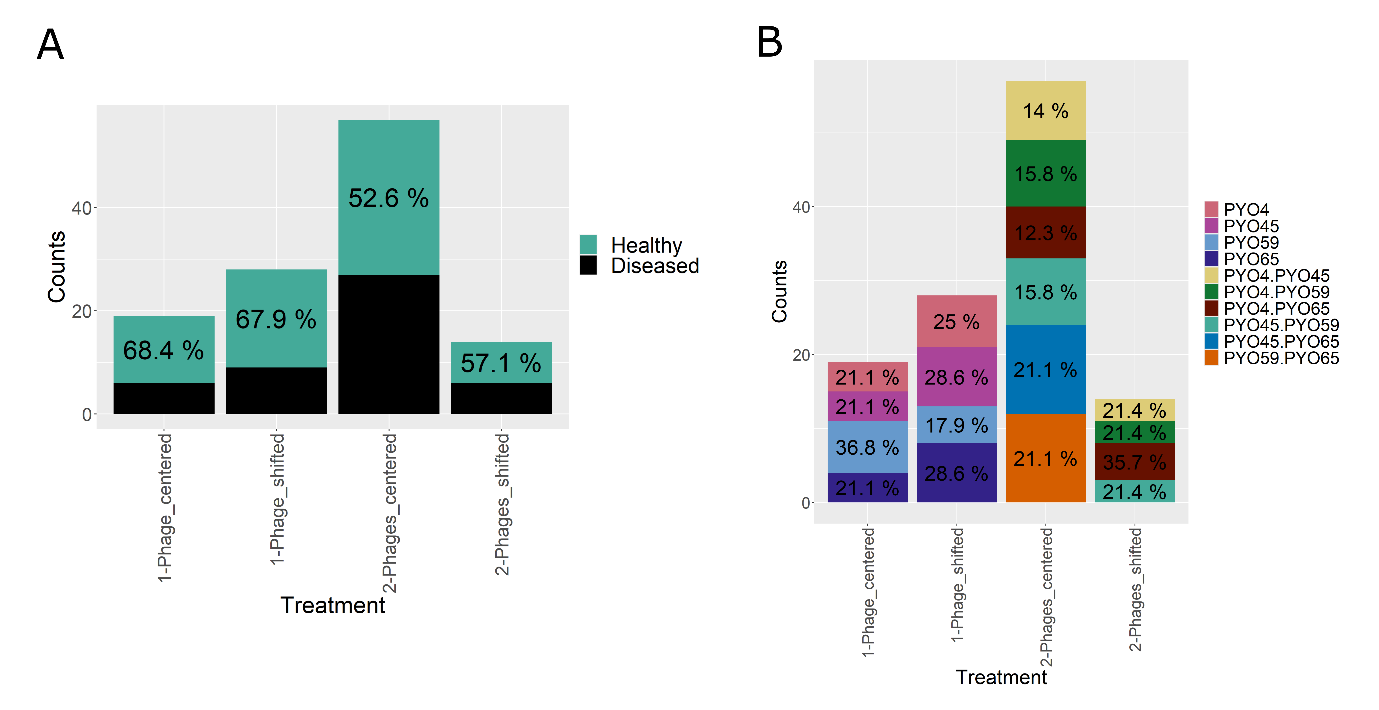


**Figure S5**. **The number and percentage of shifted bacterial communities in 1- and 2-phage treatments. A**. Number and percentage of healthy and diseased plants in the shifted and centered microbiome samples for 1- and 2-phage treatments. **B**. Number and percentage of phage treatments in the shifted and centered microbiome samples for 1- and 2-phage treatments.


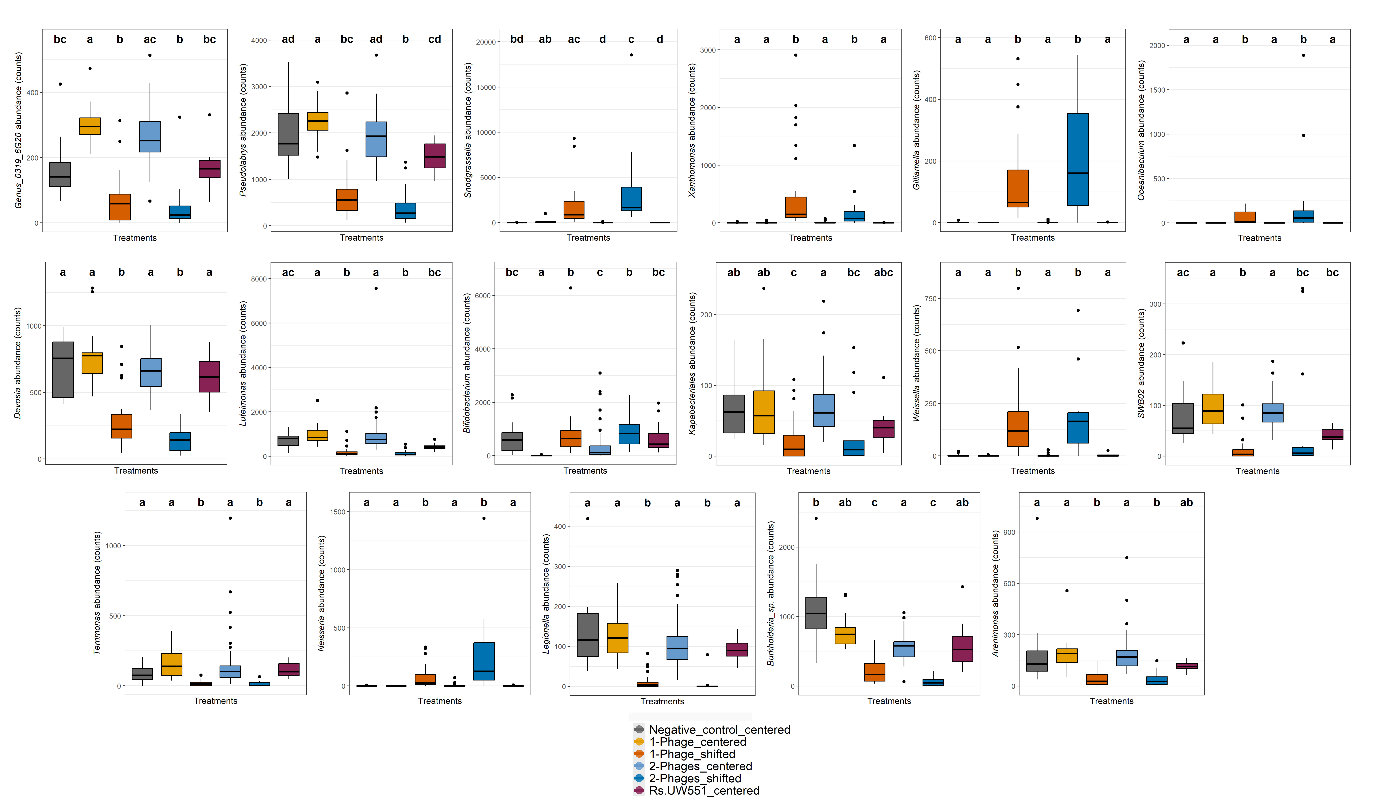


**Figure S6**. Comparison of relative abundances of 17 bacterial taxa with high Gini values between control, and ‘shifted’ and ‘centered’ 1- and 2-phage treatments (based on read counts). Genera included: 0319-6G20, *Pseudolabrys*, *Snodgrassella*, *Xanthomonas*, *Gilliamella*, *Oceanibaculum*, *Devosia*, *Luteimonas*, *Bifidobacterium*, *Kapabacteriales*, *Weissella*, *SWB02*, *Terrimonas*, *Neisseria*, *Arenimonas*, *Burkholderia-Caballeronia-Paraburkholderia* and *Legionella*.

**Supplementary tables**

**Table S1**. Number of genes in orthogroups for each of the 4 phages used in this study.

|  | PY04 | PY045 | PY059 | PY065 |
| --- | --- | --- | --- | --- |
| Number of genes | 51 | 70 | 60 | 51 |
| Number of genes in orthogroups | 51 | 6 | 7 | 51 |
| Number of unassigned genes | 0 | 64 | 53 | 0 |
| Percentage of genes in orthogroups | 100 | 8.6 | 11.7 | 100 |
| Percentage of unassigned genes | 0 | 91.4 | 88.3 | 0 |
| Number of orthogroups containing species | 49 | 5 | 7 | 49 |
| Percentage of orthogroups containing species | 96.1 | 9.8 | 13.7 | 96.1 |
| Number of species-specific orthogroups | 0 | 0 | 0 | 0 |
| Number of genes in species-specific orthogroups | 0 | 0 | 0 | 0 |
| Percentage of genes in species-specific orthogroups | 0 | 0 | 0 | 0 |

**Table S2**. Results of SYNIMA pipeline showing the orthogroups. ANGOEMEO are proteins from PY04, DGMDFAKB are proteins from PY045, HMHLAPFJ are proteins from PY059, CGDPDFMH are proteins from PY065.

**Table S3**. Bacteriophage PFU/ml in the samples of the glasshouse experiment.

**Table S4**. Quality control of the raw reads.

**Table S5**. Absolute quantification after DNA weight normalization.

**Table S6.** ASVs taxonomy.

**Table S7**. Abundance of the 2747 ASVs after rarefying.

**Table S8**. *Ralstonia*’s abundance.

**Table S9**. Bacterial wilt disease of *R. solanacearum* UW551 in tomato cv Moneymaker from 7 dpi to 22 dpi. P1=PY04, P2=PY045, P3=PY059, PY4=PY065.

**Table S10**. Alpha diversity results.

**Table S11**. Results of Random Forest analysis, soreted by Mean Gini value for each genus.
